# 16p13.11 deletion variants associated with neuropsychiatric disorders cause morphological and synaptic changes in induced pluripotent stem cell-derived neurons

**DOI:** 10.3389/fpsyt.2022.924956

**Published:** 2022-11-03

**Authors:** Elizabeth D. Buttermore, Nickesha C. Anderson, Pin-Fang Chen, Nina R. Makhortova, Kristina H. Kim, Syed M. A. Wafa, Sean Dwyer, John M. Micozzi, Kellen D. Winden, Bo Zhang, Min-Joon Han, Robin J. Kleiman, Catherine A. Brownstein, Mustafa Sahin, Joseph Gonzalez-Heydrich

**Affiliations:** ^1^Human Neuron Core, Rosamund Stone Zander Translational Neuroscience Center, Boston Children’s Hospital, Boston, MA, United States; ^2^Department of Neurology, Boston Children’s Hospital, Boston, MA, United States; ^3^Harvard Medical School Teaching Hospital, Boston, MA, United States; ^4^The Manton Center of Orphan Disease Research, Boston Children’s Hospital, Boston, MA, United States; ^5^Department of Psychiatry, Developmental Neuropsychiatry Research Program, Boston Children’s Hospital, Boston, MA, United States

**Keywords:** iPSC-derived neurons, neuropsychiatric disorders, 16p13.11 deletion, neurite morphology, synapses, *in vitro* modeling

## Abstract

16p13.11 copy number variants (CNVs) have been associated with autism, schizophrenia, psychosis, intellectual disability, and epilepsy. The majority of 16p13.11 deletions or duplications occur within three well-defined intervals, and despite growing knowledge of the functions of individual genes within these intervals, the molecular mechanisms that underlie commonly observed clinical phenotypes remain largely unknown. Patient-derived, induced pluripotent stem cells (iPSCs) provide a platform for investigating the morphological, electrophysiological, and gene-expression changes that result from 16p13.11 CNVs in human-derived neurons. Patient derived iPSCs with varying sizes of 16p13.11 deletions and familial controls were differentiated into cortical neurons for phenotypic analysis. High-content imaging and morphological analysis of patient-derived neurons demonstrated an increase in neurite branching in patients compared with controls. Whole-transcriptome sequencing revealed expression level changes in neuron development and synaptic-related gene families, suggesting a defect in synapse formation. Subsequent quantification of synapse number demonstrated increased numbers of synapses on neurons derived from early-onset patients compared to controls. The identification of common phenotypes among neurons derived from patients with overlapping 16p13.11 deletions will further assist in ascertaining common pathways and targets that could be utilized for screening drug candidates. These studies can help to improve future treatment options and clinical outcomes for 16p13.11 deletion patients.

## Introduction

Numerous neurodevelopmental disorders (NDDs) have been linked to chromosomal copy number variants (CNVs) (1–3). In particular, deletions and duplications at chromosome 16p13.11 have been associated with autism, schizophrenia, intellectual disability, and epilepsy (4–8). The region of chromosome 16p13.11 containing CNVs is divided into three main intervals. Although there are variations in the size of deletions and duplications amongst patients, identified CNVs are most frequently located inside intervals I and II (7). One study examined 3812 patients with epilepsy and found deletions greater than 100kb within the 16p13.11 locus in 23 of these patients, whereas none of the 1299 neurologically normal controls had a deletion greater than 16kb in this area (6). In another study of males with a range of neurodevelopmental conditions (57% with developmental delay), 13 out of 6595 neurodevelopmentally impaired individuals had a 16p13.11 deletion compared to none out of 4474 controls (*p* = 0.001) (9). The association between schizophrenia and 16p13.11 deletions has been reported at an odds ratio of 2.7, which increases to 3.6 in males, although neither reaches statistical significance (7). However, in this study, patients with epilepsy or developmental delay were excluded, potentially leading to the exclusion of 16p13.11 deletion patients. Other manifestations of 16p13.11 CNVs include aortic abnormalities, increased aggression, and delayed development of motor skills (10). In contrast to duplications, which commonly vary between 1.16 and 2.56Mb, 16p13.11 deletions are generally smaller, ranging between 0.81 and 1.13 Mb (10). Since reported clinical phenotypes are heterogeneous, it has been difficult to establish a disease mechanism for how CNVs in this region affect neurodevelopment.

While detailed sequencing analysis of 16p13.11 CNVs have identified genes within affected regions that are commonly associated with NDDs, the relationship between observed genetic mutations and cellular phenotypes for many of the affected genes remains unknown. A recent study using induced pluripotent stem cell (iPSC)-derived neurons found that targeting the NF_*K*_B p65 pathway was able to correct proliferation deficits caused by 16p13.11 microduplication, implicating this pathway in the pathogenesis of this syndrome (11). Despite advances in understanding the mechanisms underlying 16p13.11 microduplication, the morphological and synaptic alterations that underlie the clinical phenotypes associated with 16p13.11 deletions have not been characterized. Furthermore, identification of common cellular phenotypes between patients with different 16p13.11 deletion sizes remains largely unknown. Studies directed at elucidating common phenotypes between patients with different mutation sizes provide the opportunity to establish genotype-phenotype relationships for key cell biological features that are responsive to phenotypic screening approaches capable of dissecting the molecular basis of dysregulated pathways in patient neurons.

Using exome sequencing and microarray analyses, we identified a subset of patients with early- and young adult-onset psychosis with heterozygous deletions within chromosome 16p13.11. In this study, we derived human iPSCs from two families with patients harboring 16p13.11 deletions (probands, patients) and familial controls. One of the probands has only interval I deleted, while the other two patients, a father and son pair, have both intervals I and II deleted (4). Patient-derived iPSCs provide a platform to study the cell-autonomous effects of 16p13.11 CNVs on human neurons.

We hypothesized that loss of region I, which includes the genes *PDXDC1*, *NTAN1*, and *RRN3*, would contribute to common cell autonomous phenotypes in iPSC-derived neurons from 16p13.11 deletion patients, which may play a role in the development of clinical phenotypes. To test this hypothesis and identify specific genes and pathways affected by the 16p13.11 deletions, we generated cortical neurons and used targeted RNA sequencing to confirm that genes within the 16p13.11 region of the patients with heterozygous deletions were expressed at reduced levels when compared to controls. We also analyzed targeted RNA sequencing data from cortical neurons derived from proband and control lines and identified a set of differentially expressed genes previously associated with NDDs. Notably, protocadherins and other genes important for axonal growth, arborization and synaptic development were differentially expressed. Finally, we demonstrate that iPSC-derived neurons from patients with early- and young adult-onset psychosis display common phenotypes of increased cell body area, neurite branching, and synapse number, despite differences in the size of 16p13.11 deletion regions. Together, these data suggest that heterozygous deletion of interval I in the 16p13.11 region contributes to cell autonomous deficits in iPSC-derived cortical neurons.

## Materials and methods

### Induced pluripotent stem cell lines

The patients recruited for this study were consented through the Manton Center for Orphan Disease Research, with the approved institutional review board protocol number 10-02-0053. Informed consent was obtained from a parent if the patient was unable to do so. Two families were recruited with children who carry deletions in 16p13.11. Of the recruited participants, all 3 probands/patients are male and both controls are female. Familial male controls were not available at the time of recruitment. Patients were recruited for the study based on their 16p13.11 deletion status and other factors of sex, ethnicity, race, or age were not considered as selection criteria. The iPSC lines utilized for this project were derived from PBMCs and were reprogrammed using Sendai virus at the Harvard Stem Cell Institute iPS Core (12). 16p13.11 deletions were confirmed using chromosomal microarray from patient blood samples (Agilent CGH_SNP custom array). The iPSCs expressed the pluripotency markers NANOG, TRA1-60, OCT4, and SSEA4. iPSCs were initially derived and maintained in mTeSR-1 media (STEMCELL Technologies #85850) and later adapted to StemFlex medium (ThermoFisher #A3349401) on Geltrex (ThermoFisher #A1413301) and passaged approximately once a week with Gentle Cell Dissociation Reagent (STEMCELL Technologies #07174). iPSCs were routinely karyotyped and maintained within 10 passages out of karyotype. Passage numbers were kept similar between probands and controls and did not exceed 40 passages overall.

### Neuronal differentiation and re-plating conditions

All five iPSC lines were cultured and differentiated in parallel, with a minimum of three independent differentiation batches for each experimental assay. NGN2-induced cortical neurons were differentiated according to the protocol published by Zhang et al. in 2013 (13), with modifications described below. On differentiation day -2, iPSCs were dissociated with Accutase (Innovative Cell Technologies #AT104) and plated at 90,000 cells/cm^2^ in mTeSR-1 media supplemented with 10μM ROCK inhibitor (Y-27631, Cayman Chemical #10005583), on Geltrex-coated 12-well plates. The following day, on differentiation day -1, iPSCs were transduced with lentiviral vectors expressing NGN2, eGFP, and rtTA along with 8 μg/ml polybrene (Sigma-Aldrich #TR-1003-G). High quality lentiviral particles were produced and concentrated at the Viral Core at Boston Children’s Hospital. Plasmids used were gifts from the Thomas Sudhof laboratory, but all components can be purchased from Addgene with the following IDs: 52047, 30130, 20342. On differentiation day 0, NGN2 and eGFP expression was induced via treatment with 2μg/ml doxycycline (Millipore #324385). Cells that were positive for infection with the viral vectors were selected via treatment with 1 μg/ml puromycin (Invitrogen #ant-pr-1) for 24–48 h, depending on cell density. This selection helps to generate a relatively pure population of neurons. For the first 2 days, the following growth factors and supplements were added to the N2 medium: 10 ng/ml BDNF (Peprotech #450-02), 10 ng/ml NT3 (Peprotech #450-03), and 0.2 mg/L laminin (ThermoFisher #23017-015). Differentiating cells were then fed with B27 media containing the following growth factors and supplements every other day until differentiation day 6: 10 ng/ml BDNF, 10 ng/ml NT3, 0.2 mg/L laminin, 2 μg/ml doxycycline, and 2μM Ara-C (Sigma-Aldrich #C1768). Contrary to the originally published protocol, on day 2, no mouse glial cells were added. Instead, astrocyte-conditioned media was collected from cultures of iPSC-derived astrocytes (NCardia Astro.4U) and neurons were cultured in this human-astrocyte-conditioned media along with growth factors and supplements after day 6 to avoid astrocyte signals in the RNA and protein analysis. Neurons were matured until day 14 prior to being collected for RNA or protein analysis. For morphological analyses, NGN2 neurons were dissociated on day 6 with ≥16 units/mL papain (Worthington #LK003178) supplemented with ≥166 Kunits/mL DNase I (Worthington #LK003172) for 20 min at 37°C.

For neurite morphology assays, neurons were re-plated at differentiation day 6 onto 96-well plates (VWR #82050-748) that were coated with poly-d-lysine (PDL) (Sigma-Aldrich #P6407) overnight followed by three washes with sterile diH_2_O and laminin (Life Technologies #23017-015) coating overnight. Neurons were plated at a density of 10,000 neurons per well and were fixed with 4% paraformaldehyde (PFA) (ThermoFisher #28908) 24 h after plating by adding 8% PFA in a 50:50 mix with media remaining in the well so that the neurons were never exposed to air prior to fixation. Neuron cell counts were obtained using trypan blue stain mixed 1:1 with a small aliquot of cells and counted on a manual hemocytometer.

For synapse analysis, neurons were re-plated onto PDL and laminin-coated 96-well plates on day 6 at a density of 10,000 neurons per well and were co-cultured with 1,500 iPSC-derived astrocytes (NCardia Astro.4U) per well. Astrocytes were co-cultured with neurons directly from frozen vials and leftover astrocytes were plated onto PDL-coated T75 flasks (Westnet #354537) for conditioned media collection. Neuron/glia co-cultures were matured until day 24 when they were fixed with ice-cold methanol at −20C. Following fixation for 10 min at −20C, plates were warmed to room temperature in methanol before three washes were completed with 1XPBS.

### Immunocytochemistry

For all immunocytochemistry, cells were blocked for 1 h with a solution of 5% normal goat serum (Sigma-Aldrich #G9023) and 0.1% Triton-X (ThermoFisher #AC215682500) in 1X PBS (Life Technologies #14190250). For iPSC immunostaining, additional permeabilization was achieved by an additional wash with 0.5% Triton-X prior to blocking. Following blocking, primary antibodies were diluted in blocking buffer and incubated on the cells overnight at 4°C. Primary antibodies used include anti-β3-tubulin (Sigma Aldrich #T8660), anti-β3-tubulin (Novus Biologicals #NB100-1612), anti-MAP2 (Abcam #ab5392), anti-Synapsin-1 (Millipore Sigma #AB1543P), anti-PSD95 (Antibodies Incorporated #75-028 NeuroMab), anti-Synaptophysin (Millipore Sigma #S5768), anti-NR2B (Millipore Sigma #06-600), anti-Nanog (Abcam #ab109250), anti-TRA-1-60 (ThermoFisher #411000), anti-OCT4 (Abcam #ab181557), and anti-SSEA4 (ThermoFisher #414000). The next morning, cells were washed three times with 1X PBS prior to incubation at room temperature for 1 h with secondary antibodies that were diluted in blocking buffer. Secondary antibodies used include goat anti-mouse Alexa Fluor 568 (Invitrogen by ThermoFisher #A11031), goat anti-chicken Alexa Fluor 488 (Invitrogen by ThermoFisher #A11039), goat anti-rabbit Alexa Fluor 647 (Invitrogen by ThermoFisher #21245), goat anti-mouse Alexa Fluor 488 (Invitrogen by ThermoFisher #A11029), and goat anti-rabbit Alexa Fluor 568 (Invitrogen by ThermoFisher #A11036). Included in the secondary antibody mixture was Hoechst 33258 (Invitrogen #H3569) to counterstain the nuclei. Cells were then washed three times with 1X PBS and stored at 4°C in 1X PBS with 0.02% sodium azide (Sigma Aldrich #S2002) prior to imaging. iPSC colonies were imaged on the EVOS FL imaging system (ThermoFisher #AMF4300) at 10X magnification, and neurons were imaged on the ImageXpress Micro-Confocal automated microscope (Molecular Devices).

### Automated microscopy

All neurons were imaged on the ImageXpressMicro-confocal automated microscope from Molecular Devices. Exposure and focus settings were established on control wells and utilized across all wells of the same staining conditions. To capture neuron morphology, the 10X objective was utilized. To capture synapses, the 20X objective was used. For each line and endpoint, 5–6 wells were imaged in each batch, with 9 fields per well. A minimum of three batches of replicate differentiations were completed with every line differentiated simultaneously in each batch. Following imaging, analysis was completed using the MetaXpress software.

### Image analysis

For neurite morphology assays the Neuronal Profiler Application Module was used to quantify neuron cell count, soma size, as well as neurite length, number, and branching. Synapse analysis was completed using the Custom Module Editor (CME), in which the following steps were combined to quantify synapses: “neurite outgrowth application” within CME was used to identify neuronal cell bodies and neurites, “find blobs” was used to identify Synapsin-1 puncta, “find blobs” was used to identify PSD95 puncta, “logical operations” was used to overlay Synapsin-1 and PSD95 masks to create a “synapse” mask, “logical operations” was used to overlay the synapse mask with the neurite mask to quantify synapses on neurites. For each morphological feature, the average control value for each batch was used to normalize control and proband values so that batch to batch differences in neurite outgrowth could be controlled for. The proband or patient values were compared with the familial control neurons within the same family. Adobe Photoshop was utilized to crop images and change pseudo-colors for overlay purposes during figure construction. All changes to image brightness and contrast were conducted equally across patient and control images.

### Transcriptional profiling

At day 14, neurons were collected, and total RNA was isolated (Clontech #740971.50). Isolated total RNA was diluted to 0.95ng/μl and 10.5μl was loaded in each reverse transcription reaction with the SuperScript VILO cDNA Synthesis Kit (ThermoFisher #11754050). Reverse transcription was completed in a thermocycler with the following temperature settings: 42°C 30 min, 85°C for 5 min, 10°C Hold. After reverse transcription, libraries were prepped using the Ion Chef (ThermoFisher Cat#4484177) and the Ion AmpliSeq Transcriptome Human Gene Expression Panel, Chef-Ready Kit (ThermoFisher Cat#A31446). The AmpliSeq Gene Expression Panel includes one primer pair for almost every gene in the transcriptome, allowing for amplification of a single 100bp amplicon for each gene to be sequenced. Libraries from 8 samples were pooled together, templated, and loaded on the Ion 540 Chip (ThermoFisher Cat#A27765) using the Ion Chef and the Ion 540 Kit Chef (ThermoFisher Cat#A27759). After templated samples were loaded onto the 540 Chips, each Chip was inserted into the Ion Torrent S5 sequencer (ThermoFisher Cat#A27212), and 500 flows were applied for each sequencing run. The second Chip was stored at 4°C during the first sequencing run and removed to room temperature for 30 min prior to running the second Chip. Read mapping and preliminary data analysis was completed with the AmpliSeq Plugin available on the ThermoFisher S5 Torrent server. CHP files were loaded into the Transcriptome Analysis Console (TAC) Software from ThermoFisher to complete differential gene expression analysis of the probands versus controls, with 1.2-fold change in expression as cutoff and p-values corrected for multiple comparisons using FDR. Gene ontology analysis was completed using DAVID.^1^ Genes not shown in the paper were absent due to one of two potential factors: (1) absence of the gene from our transcriptome primer pool, or (2) low expression of the transcript.

For qPCR, isolated RNA was converted into cDNA using SuperScript IV VILO (ThermoFisher #11756050). PCR reactions were assembled using TaqMan Gene Expression Mastermix (ThermoFisher #4369016) and the following TaqMan probes: Hs02786624g1 GAPDH TaqMan, Hs02800695_m1 HPRT1 TaqMan, Hs00415811_m1 PDXDC1 TaqMan, Hs04964431_gH PKD1P1/NPIP TaqMan. PCR was run on an Applied Biosystems 7500 fast real-time PCR system. We used GAPDH and HPRT1 as the loading control for each gene and fold changes were calculated using the ΔΔCt method.

### Statistical tests

One-way and two-way analysis of variance (ANOVA) was performed to compare means between unmatched groups of data (batches and lines; a proband and a control within a family). Normality of groups of data was tested using D’Agostino and Pearson test and variance similarity was tested using Bartlett’s test for equal variances. Otherwise, a Kruskal-Wallis test or a Mann-Whitney test was performed. There were no statistical methods used to predetermine sample size. The experiments were not randomized. Statistical analyses were conducted using Prism (GraphPad) and R (Version 3.6.1, R Foundation for Statistical Computing, Vienna, Austria).

## Results

### Induced pluripotent stem cell and neuronal characterization

**FIGURE 1:**
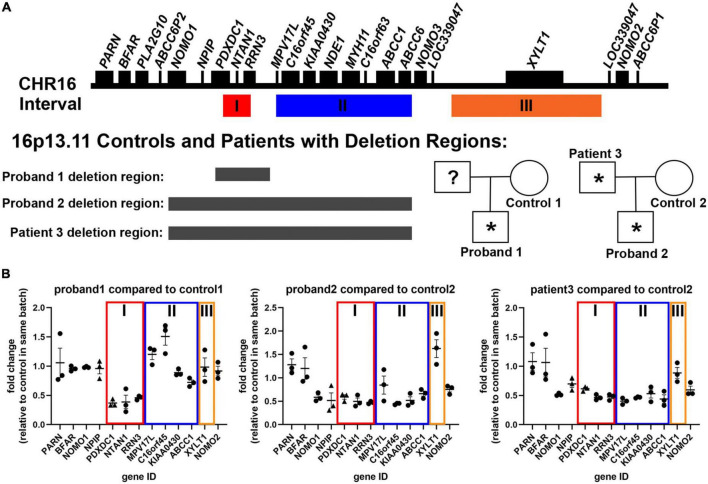
Description of patient samples recruited to study 16p13.11 deletion. **(A)** Representation of genes present in the 16p13.11 region, including intervals I, II, and III, and designation for inheritance pedigree and deletion regions found in probands 1 and 2 and patient 3. **(B)** AmpliSeq (circles) and qPCR (triangles) results from day 14 iPSC-derived cortical neurons showing changes in gene expression levels of genes in the 16p13.11 region in all three patients. Data are shown as ± SEM, *n* = 3 differentiations.

The iPSCs used for this study were collected from two families affected by psychosis. The proband from the first family was previously described (4). Proband 1 has a microdeletion of interval I 16p13.11 (Figure 1). Proband 1 had delayed development of motor coordination, verbal reasoning, and non-verbal reasoning and began having auditory, visual, and tactile hallucinations at the age of 6 (4). Control iPSCs were generated from proband 1’s mother (control 1), who does not have a chromosomal deletion within the 16p13.11 region but has a history of depression and anxiety. Proband 1’s father has probable schizophrenia, but was unavailable for testing, so the CNV is either *de novo* or inherited from the father (4).

The second family has two affected patients, father and son, each with a deletion that spans Intervals I and II of 16p13.11 (Figure 1). The deletion region for proband 2 and patient 3 are different than proband 1 and include a few genes upstream of the 16p13.11 region, including *NOMO1*. Proband 2, the son in family 2, also has a duplication at 18q22.1 of unknown significance (93kb not reported in currently available literature/database resources and encompassing no genes associated with any syndrome) and a maternally inherited mutation in *GRIN2A* (one of the subunits of NMDA receptors) that has not been previously reported. Proband 2 has Autism Spectrum Disorder (ASD) with young adult-onset schizophrenia (age 22 years), along with language delay, intellectual disability, and epilepsy, while his father, patient 3, developed bipolar I disorder with psychosis for the first time at age 46 years in the context of treatment with high dose mixed amphetamine salts (Adderall 80 mg/day). He has a history of congenital heart disease and had seizures as a child. Both proband 2 and patient 3 have required long term treatment with antipsychotic medication (clozapine and risperidone, respectively). Control 2 is the mother of proband 2, who has the same mutation in *GRIN2A*, but shows no observable clinical psychological or neurological abnormalities. Proband 2 and his mother, control 2, have macrocephaly (head circumference > 98%), and this runs in control 2’s family. Importantly, despite differences in the size of the 16p13.11 deletion, and the age of psychosis onset, all 3 probands displayed psychotic symptoms. Further, none of the deletions found in either proband or patient 3 encompasses interval III, which only contains the single gene, *XYLT1* (Figure 1).

The iPSCs generated from each patient or control were tested for expression of the pluripotency markers OCT4, TRA1-60, NANOG, and SSEA4 and karyotyped prior to beginning work with the lines (Supplementary Figures 1, 2). Master banks of each iPSC lines were prepared, and fresh vials were thawed for use in experiments after 5-10 passages.

**FIGURE 2:**
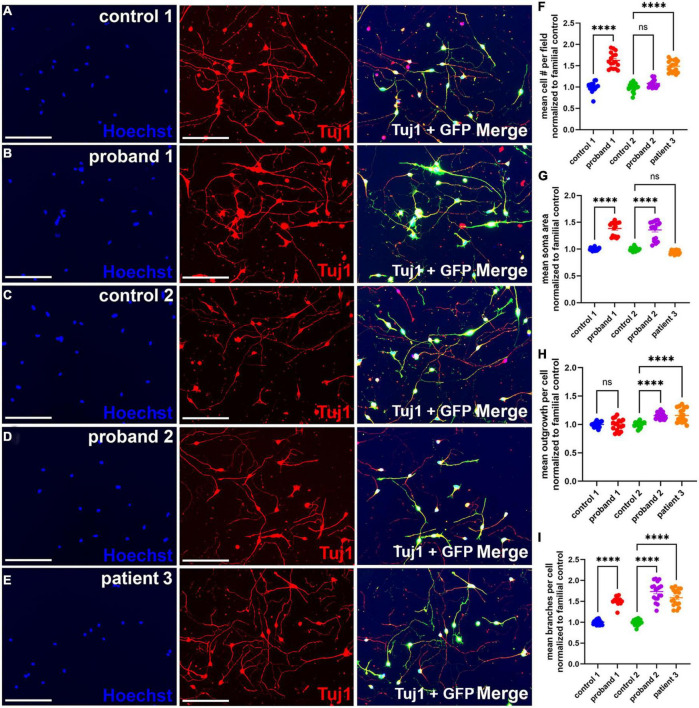
Increased neurite branching in 16p13.11 deletion iPSC-derived NGN2 neurons. **(A–E)** Representative images of day 7 16p13.11 proband and control iPSC-derived neurons immunostained with Hoechst (blue), with GFP signal from lentiviral induction (green), and Tuj1 (red). All images taken at 10X. For all lines, 9 fields were imaged per well, 5 wells per line, and 5 separate differentiations were compiled for the subsequent quantification. **(F)** Quantification of the mean neuronal cell number per field revealed an increase in 2 of 3 patients. **(G)** Quantification of the mean soma area revealed an increase in 2 of 3 patients. **(H)** Quantification of mean outgrowth per cell revealed an increase in 2 of 3 patients. **(I)** Quantification of mean branches per cell revealed an increase in all three patients. Scale bars = 300 μm. In panels **(F–I)** data are shown as ± SEM, each data point represents a mean well value averaged from 9 fields per well across 3 differentiations, n.s. = not significant, ****p < 0.0001. Pseudo-coloring was applied to allow for merged images.

In order to study the cell autonomous effects of these deletions on cortical neurons, we utilized the Neurogenin-2 (NGN2) induction protocol to generate neurons (13). NGN2 neural induction was initiated at day 0 using doxycycline treatment, which was removed after day 6 (see schematic in Supplementary Figure 3). Neurons were collected for RNA analysis at differentiation day 14. Analysis of targeted RNA sequencing data confirmed neuronal identity based on expression of neuronal genes (*TUBB3*, *MAP2, NCAM1*), as well as downregulation of pluripotency genes (*NANOG, FUT4, SOX2*), and neural progenitor genes (*PAX6, SOX1*) (Supplementary Figure 3). The neural progenitor gene, *NESTIN* (*NES*), was still expressed at low levels in the day 14 neurons (Supplementary Figure 3). High content image analysis of TUJ1 and MAP2 at day7 of differentiation also revealed the efficient production of neurons at this early time point in the differentiation (Supplementary Figure 3). Interestingly, proband 1 produced more MAP2-positive neurons than control 1, as quantified by both high content imaging and transcriptomics. Neurons also displayed distinctive neuronal morphology, with rounded soma and multiple neurites (Figure 2). Together, these data reveal that control and 16p13.11 deletion iPSCs were all capable of differentiating into NGN2-cortical neurons. We also utilized our transcriptomic data to confirm downregulation of genes within interval I for proband 1, and within intervals I&II for proband 2, and patient 3 (Figure 1).

### Neuronal morphological effects of 16p13.11 deletion

The third gene in 16p13.11 interval I, *RRN3*, produces a protein that plays an important role in neurite outgrowth in rodent hippocampal neurons (14). Therefore, we hypothesized that deletions encompassing *RRN3* would similarly affect neurite outgrowth in proband iPSC-derived neurons compared to controls. To test this hypothesis, neurons were re-plated at differentiation day 6 and allowed to grow for 24 h prior to fixation, staining with β3 tubulin (Tuj1) and analysis for soma and neurite morphology (Figure 2). Examples of neurite masks on control and proband neurons reveal neuron soma selection, neurite tracing for individual neurons, and exclusion of non-neurons (Supplementary Figure 4). Detailed analyses of iPSC-derived neurons revealed an increase in the mean number of cells per field in proband 1 and patient 3, but not proband 2, compared to familial controls (Figure 2F). There was also an increase in mean soma area in probands 1 and 2, but not patient 3, which showed a significant decrease in soma area (Figure 2G). Surprisingly, we observed an increase in mean outgrowth per cell for proband 2 and patient 3 compared to their familial control, but we did not observe a similar increase in the mean outgrowth for proband 1. Interestingly, we also found a significant increase in mean branches per cell in all three patients, compared with familial controls (Figure 2I). Overall, these results indicate that deletion of the 16p13.11 region affects neuron morphology.

### Transcriptional changes due to 16p13.11 deletion

**FIGURE 3:**
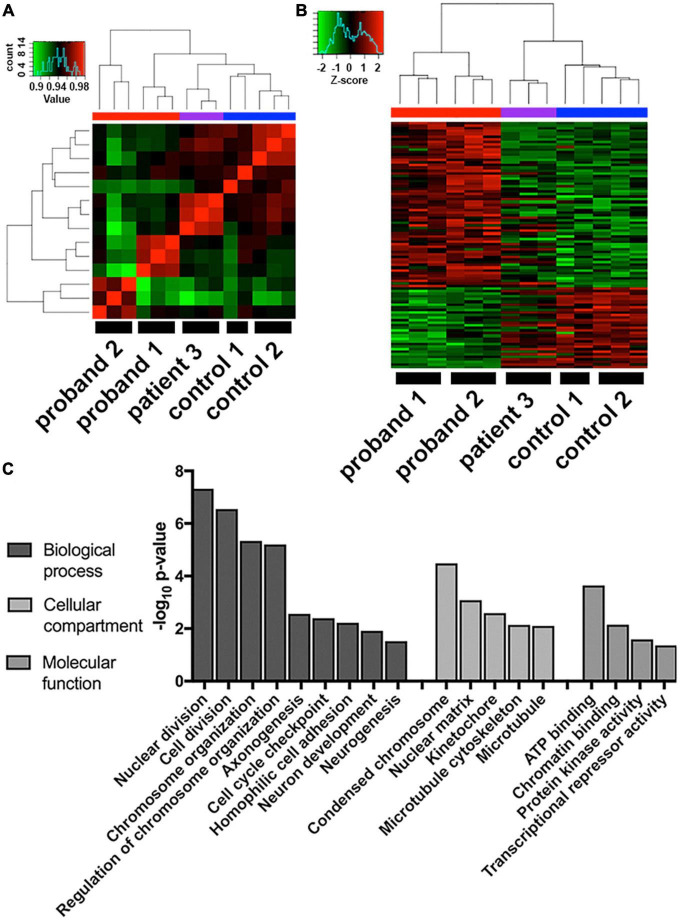
Transcriptome analysis of 16p13.11 deletion iPSC-derived neurons. **(A)** Comparison of whole transcriptome sequences from day 14 iPSC-derived neurons across the 5 lines revealed grouping of patient 3 closer to the two control lines than the two proband lines. **(B)** Comparison of the top 2-fold changed genes from the gene expression data across the 5 lines revealed probands 1-2 and controls 1-2 grouping with each other and patient 3 falling somewhere in the middle. **(C)** Gene ontology of the whole transcriptome data describing which cellular pathways were most changed in proband 1-2 neurons compared to control 1-2 neurons.

To examine which molecular pathways were altered by deletions within the 16p13.11 region that could explain the morphological changes observed, we utilized an unbiased AmpliSeq approach to compare gene expression levels of 20,000 + transcripts across three technical replicates of NGN2 neurons from all 5 iPSC lines. Cluster analysis of the full 20,000 + transcripts revealed that patient 3, the father from our second family, clusters more closely with the two control lines, than with the two probands (Figure 3A). Furthermore, when examining the transcripts that were 1.2-fold up- or down-regulated in the probands, versus familial controls, patient 3 again clusters more closely with the controls than the probands (Figure 3B). Importantly, unsupervised clustering shows that the three samples from independent differentiation batches within each individual most closely associate with one another, suggesting that the individual’s genotype is a bigger factor for the transcriptomic signature than batch-to-batch differentiation variability. Given the difference in patient 3’s transcriptome and since patient 3 had a much later onset of psychosis that was triggered by medication, his samples were excluded from further transcriptomic analysis. To eliminate gender-specific bias, given both probands are male and both controls are female, a previously published list of gender-specific genes (15) were eliminated from the top-regulated gene list. The complete list of genes that were 1.2-fold up or down-regulated between probands 1 and 2 and familial controls is included in Supplementary Table 1. Gene ontology analysis of the differentially expressed genes revealed that these genes were enriched in pathways related to cell division, chromosome organization, and other cell cycle-related pathways (Figure 3C). In addition, we found changes in axonogenesis, neuron development, neurogenesis, microtubule cytoskeleton, protein kinase activity, and transcriptional repressor activity, which could all lead to changes in neuronal morphology. We also observed multiple members of the protocadherin family were disrupted in our 16p13.11 deletion iPSC-derived neurons compared to their familial controls. Given that PCDH19 has been implicated in pathology resulting from 16p13.11 duplication, we sought to further evaluate potential functional phenotypes impacted by protocadherin dysregulation in the deletion patients (16). Additionally, we examined expression of synaptic markers and found that several synapse-related genes were upregulated in proband 1 compared to control 1, but no change was detected in proband 2 or patient 3 compared to control 2 (Supplementary Figure 5).

### Changes in synapse formation in 16p13.11 induced pluripotent stem cell-derived neurons

**FIGURE 4:**
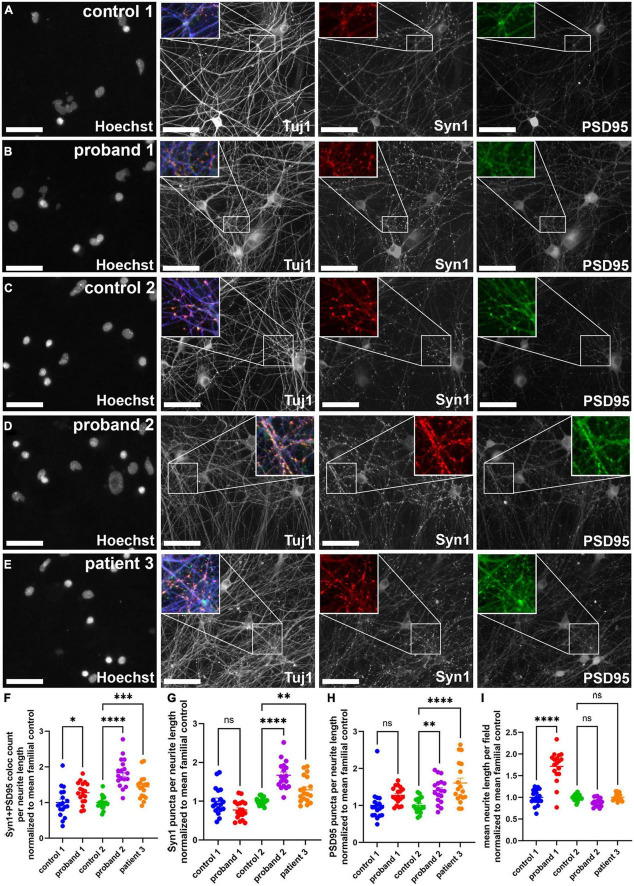
Presumptive synapse count was increased in 16p13.11 deletion long-term cultures. **(A,B)** Representative images of control 1 **(A)** and proband 1 **(B)** long-term day 24 NGN2 neurons co-cultured with astrocytes and immunostained for Tuj1, Synapsin 1 (Syn1), and PSD95. Representative images of control 2 **(C)**, proband 2 **(D)**, and patient 3 **(E)** long-term day 24 NGN2 neurons co-cultured with astrocytes and immunostained for Tuj1, Synapsin 1 (Syn1), and PSD95. All images were captured at 20X. **(F)** Quantification of average Syn1 + PSD95 colocalized puncta per neurite length. **(G)** Quantification of Syn1 puncta per neurite length. **(H)** Quantification of PSD95 puncta per neurite length. **(I)** Quantification of mean neurite length per field. Scale bars = 50 μm. In panels **(F–I)** data are shown as ± SEM, each data point represents a mean well value averaged from 9 fields per well across 5 differentiations, n.s. = not significant, *p < 0.05, **p < 0.01, ***p < 0.001. Pseudo-coloring was applied to allow for merged images.

As has been reviewed in the literature, changes in synapse function and number are linked to many neurological disorders, including psychiatric disorders (17). Since changes in synapse number could affect neuronal circuit formation and function, we decided to examine whether synapse number was altered in 16p13.11 deletion neuron cultures, compared to those from controls. We co-cultured cortical neurons from each of the five iPSC lines with healthy control iPSC-derived astrocytes on day 6 of differentiation and allowed the neuronal networks to mature for 16 days. At this time, cultures were fixed and stained for synaptic markers, including Synapsin-1 and PSD95 (Figure 4). An example of the synapse quantification algorithm shows identification of cell bodies, neurites, and synapsin1 puncta (Supplementary Figure 6). Quantification of the number of Synapsin-1/PSD95 co-localized puncta revealed an increase in presumptive synapse number per neurite length in all three patients, compared with controls (Figure 4). Interestingly, both PSD95 puncta per neurite length and Synapsin1 puncta per neurite length were significantly increased in proband 2 and patient 3 compared to control 2, but no change was seen in these individual synapse markers for proband 1 compared to control 1 (Figure 4). There was also a striking increase in neurite outgrowth in proband 1 compared to control 1 (Figure 4), however, this could be due to the lower level of neurite outgrowth seen in control 1 compared to the other lines (Supplementary Figure 7). Since NGN2-induced neurons are predominantly glutamatergic, we also examined whether presumptive glutamatergic synapses were altered by quantifying colocalized puncta stained with antibodies against Synaptophysin (SYP) and the glutamate receptor subunit NR2B (Supplementary Figure 8). Interestingly, we did not see a significant difference in NR2B-containing presumptive synapses, which is at odds with the differences found in SYN1 + PSD95 positive presumptive synapses.

## Discussion

While 16p13.11 deletions have been associated with epilepsy and intellectual disabilities, the links between 16p13.11 deletions and early onset psychosis are just beginning to be understood. Furthermore, little is known about the cell autonomous effects of these deletions on cortical neurons. Elucidating the effects of these deletions on patient neurons will help researchers and clinicians learn where therapeutic interventions may be possible. Here, we utilized iPSCs generated from two families with 16p13.11 deletions to examine the neuronal morphological and molecular consequences of these deletions. We hypothesized that common deletions within the 16p13.11 region would result in common neuronal deficits. Our studies revealed changes in neurite morphology, with an increase in neurite branching in 16p13.11 deletion neurons, compared with control neurons. Transcriptome analysis revealed changes in axonogenesis, neuron development, microtubule cytoskeleton, and protein kinase activity, all of which could affect neurite branching *in vitro*. Lastly, co-cultures of 16p13.11 deletion neurons with astrocytes revealed an increase in the numbers of synapses stained with SYN1 and PSD95. While these changes may be due to loss of the genes discussed below, it should be noted that there are less well characterized genes, including potential pseudogenes, in this region that may also contribute to phenotypes.

There is little known about the cell and molecular consequences of 16p13.11 deletions during neuronal development. Previous reports indicate no gross changes in cortical layer lamination or neuronal morphology upon examination of temporal lobe resections collected from 16p13.11 deletion patients with epilepsy (18). These patients had small deletions in region II of 16p13.11, which contains NudE nuclear distribution gene homolog 1 (*NDE1*). Many reports have documented the increased susceptibility for epilepsy in patients with 16p13.11 deletions (6, 10, 19, 20). Within the 16p13.11 region, *NDE1* has been proposed as the most likely candidate for epilepsy, as well as other neuropsychiatric symptoms (6, 19, 21–24). NDE1 is localized to the centrosome, where it interacts with Lissencephaly-1 and dynein and plays a critical role in microtubule organization, mitosis, and neuronal migration (25–30). Thus, loss of *NDE1* in proband 2 may be responsible for this patient’s seizures. Further, patient 3 also has loss of *NDE1* with a history of childhood seizures.

Since all three patient deletions include 16p13.11 interval I, we hypothesized that haploinsufficiency of interval I genes would contribute to common neuronal phenotypes found across all three patient lines. We further hypothesized that 16p13.11 deletion neurons would have deficits in neurite outgrowth due to loss of RRN3, based on phenotypic analysis of cultured *Rrn3* knockout rodent hippocampal neurons (14). Interestingly, we did not observe a decrease in neurite outgrowth in our patient derived neurons, but rather an increase in neurite branching. One potential explanation is that the loss of multiple genes in this region interact in a way that compensates for loss of RRN3, alternatively neurite outgrowth may be regulated differently between rodent and human neurons. Our transcriptome analysis found changes in genes related to the microtubule cytoskeleton, which is critical for neurite outgrowth and branching (31). In order to elucidate mechanisms responsible for the 16p13.11 deletion branching deficits, future studies should focus on determining the timeframe when branching changes occur and if they can be pharmacologically rescued by tapping into developmental pathways.

Additionally, we found that soma size was increased in probands 1 and 2, but not patient 3. Soma size differences can be caused by pathways that control cell growth, such as the PI3K/mTOR pathway, as described in *TSC2-*deficient neurons (32). RRN3 is an initiation factor for Pol1-mediated transcription and functions within the mTOR signaling pathway to regulate protein synthesis and growth (33–36). Loss of RRN3 could contribute to dysregulation of protein synthesis, due to its feedback signaling on the mTOR pathway. When cells are healthy, mTOR signaling inhibits ribosomal RNA transcription to control protein synthesis levels (35). This inhibition signals to a feedback loop that decreases mTOR activity, alleviating the brake on ribosomal RNA transcription (37). If ribosomal RNA transcription is artificially inhibited with loss of RRN3, then the feedback loop to decrease mTOR activity is broken. Soma size of iPSC-derived neurons can also be affected by differentiation efficiency. Neural progenitors and immature neurons tend to have larger cell body sizes, which decreases as neurons mature (38). Therefore, there might be deficits in neuronal differentiation or maturation in 16p13.11 deletion neurons, which is corroborated by changes in cell division, chromosome organization, and other cell cycle-related pathways found in our transcriptome data. We also found transcriptional changes in pathways associated with axonogenesis, neuron development, neurogenesis, microtubule cytoskeleton, protein kinase activity, and transcriptional repressor activity. To determine whether these changes reflect deficits in differentiation efficiency versus neurite outgrowth, patterning-based directed neuron differentiation approaches could be used. By using small molecules to mimic neuronal development, directed differentiations allow for investigation into how stages of neuronal development are altered by patient-specific mutations.

Our data revealed an increase in presumptive synapse number stained with SYN1 and PSD95 in 16p13.11 deletion neuronal cultures, compared with controls. The difference seen in proband 1 compared to control 1 could be due to the difference in neurite outgrowth even though synapse number was normalized to neurite length. It is possible that the reduced neurite outgrowth seen in control 1 long-term cultures compared to the other lines led to reduced neuronal communication and thus a reduction in synapse stability over time. Interestingly, we did not see a difference in NR2B-containing synapses. Future studies that utilize additional synaptic markers as well as more clones per line and newly recruited patient-control pairs may help better understand these findings. The changes in synapse number in patient neurons is something we hypothesized may occur in these cultures, in part due to the changes in protocadherin family protein expression level, which are known to play an important role in synapse formation (39). In addition, examination of postmortem tissue from patients with neuropsychiatric disease revealed altered expression of dendritic spine-related proteins, NMDA receptors, AMPA receptors, and proteins important for clustering these receptors, such as PSD-95, suggesting changes in synaptic plasticity are present (40–45). Protein turnover is essential for synaptic plasticity (46–50) and mutations within genes in the ubiquitin proteasome system have been found in patients with schizophrenia (51, 52). Further, components of the ubiquitin proteasome system are localized to the synapse and when they are pharmacologically or genetically perturbed, there is protein accumulation within the presynaptic terminal of the synapse (46). Importantly, *NTAN1*, in region I of 16p13.11, is an important component of the N-end rule pathway, which is responsible for targeting proteins for proteasome degradation (53). One example of a synaptic protein that is targeted by the N-end rule is α-Synuclein (54). α-Synuclein is a presynaptic protein with several roles at the synapse, including membrane remodeling and regulation of synaptic vesicles (55). The increase in synapses found in 16p13.11 deletion cultures could be due to loss of NTAN1, resulting in decreased protein turnover in synapses, allowing for stability of synapses. Interestingly, some *Ntan1* knockout mice have shown improved spatial learning and changes in synaptic plasticity (56), while others have shown deficits in social exploration and spatial memory (57). The timing of behavioral testing affected whether deficits or strengthening of learning and memory were found in *Ntan1* knockout mice (58). These studies suggest that animal models of *Ntan1* loss recapitulate some of the cognitive domains that are affected in patients with schizophrenia, including altered socialization and impairment of working memory (59, 60).

The last gene in interval I of 16p13.11, *PDXDC1*, may function to metabolize catecholamine neurotransmitters and has been found to modulate pre-pulse inhibition of acoustic startle in mouse models (61). Pre-pulse inhibition is altered in patients with schizophrenia and modulation of catecholamine neurotransmission, especially that of dopamine, is used to treat schizophrenia, suggesting that PDXDC1 is an important gene to examine in future studies, especially in iPSC-derived catecholaminergic neurons.

Altogether, this work offers insight into the cellular and molecular consequences of 16p13.11 deletions. The authors also note a limitation with the characterization of male patient and female control neurons. While there are clearly transcriptomic differences that are due to the male versus female genetic background, it is not known how these differences may contribute to the phenotypes detected. Another limitation of the study was the lack of isogenic controls due to the large deletion sizes and the technical difficulties with recapitulating or rescuing such large deletions. In lieu of isogenic pairs, future studies will benefit from increased cohort sizes. Future studies should also focus on the functional consequences of the presumptive increased synapse count. Mixed glutamatergic/GABAergic cultures should be examined to see if imbalances such as this are specific to one subtype of synapse and to help us better understand how circuit imbalances may occur in patients with these deletions. Finally, examination of phenotypes in GABAergic and dopaminergic neurons will be critical, as these neurons are clearly dysfunctional in patients with neuropsychiatric disorders. The data presented here offers a first glimpse at the cell autonomous consequences of 16p13.11 deletion in glutamatergic cortical neurons, which can now be used to develop assays for phenotypic screening to identify novel therapeutic targets for this patient population.

## Data availability statement

The datasets presented in this study can be found in online repositories. The names of the repository/repositories and accession number(s) can be found below: https://www.ncbi.nlm.nih.gov/, GSE208574, https://www.ncbi.nlm.nih.gov/, GSM6351527, https://www.ncbi.nlm.nih.gov/, GSM6351528, https://www.ncbi.nlm.nih.gov/, GSM6351529, https://www.ncbi.nlm.nih.gov/, GSM6351530, https://www.ncbi.nlm.nih.gov/, GSM6351531, https://www.ncbi.nlm.nih.gov/, GSM6351532, https://www.ncbi.nlm.nih.gov/, GSM6351533, https://www.ncbi.nlm.nih.gov/, GSM6351534, https://www.ncbi.nlm.nih.gov/, GSM6351535, https://www.ncbi.nlm.nih.gov/, GSM6351536, https://www.ncbi.nlm.nih.gov/, GSM6351537, https://www.ncbi.nlm.nih.gov/, GSM6351538, https://www.ncbi.nlm.nih.gov/, GSM6351539, https://www.ncbi.nlm.nih.gov/, GSM6351540, and https://www.ncbi.nlm.nih.gov/, GSM6351541.

## Ethics statement

The studies involving human participants were reviewed and approved by Boston Children’s Hospital IRB The Manton Center for Orphan Disease Research Gene Discovery Core Protocol #10-02-0053. Written informed consent to participate in this study was provided by the participants’ legal guardian/next of kin. Written informed consent was obtained from the individual(s), and minor(s)’ legal guardian/next of kin, for the publication of any potentially identifiable images or data included in this article.

## Author contributions

EB designed and conducted experiments, analyzed and interpreted results, and wrote the manuscript. NA conducted experiments and wrote the manuscript. P-FC and M-JH designed and conducted experiments. NM conducted experiments and data analysis. KK, SW, SD, and JM executed experiments. KW completed gene ontology analysis and interpretation. BZ completed statistical analysis for data collected. RK designed experiments, oversaw project direction, and data interpretation. CB contributed to the design of the study and interpretation of results. MS oversaw project direction and data interpretation. JG-H provided access to the patient population, oversaw project direction, and data interpretation. All authors contributed to the article and approved the submitted version.

## Footnotes

1
https://david.ncifcrf.gov

## References

[1] Cooper GM Coe BP Girirajan S Rosenfeld JA Vu TH Baker C A copy number variation morbidity map of developmental delay. *Nat Genet.* (2011) 43:838–46. 21841781 10.1038/ng.909 PMC3171215

[2] Gilissen C Hehir-Kwa JY Thung DT van de Vorst M van Bon BW Willemsen MH Genome sequencing identifies major causes of severe intellectual disability. *Nature.* (2014) 511:344–7. 24896178 10.1038/nature13394

[3] Pinto D Pagnamenta AT Klei L Anney R Merico D Regan R Functional impact of global rare copy number variation in autism spectrum disorders. *Nature.* (2010) 466:368–72. 20531469 10.1038/nature09146 PMC3021798

[4] Brownstein CA Kleiman RJ Engle EC Towne MC D’Angelo EJ Yu TW Overlapping 16p13.11 deletion and gain of copies variations associated wiath childhood onset psychosis include genes with mechanistic implications for autism associated pathways: two case reports. *Am J Med Genet A.* (2016) 170A:1165–73. 10.1002/ajmg.a.37595 26887912 PMC4833544

[5] Hannes FD Sharp AJ Mefford HC de Ravel T Ruivenkamp CA Breuning MH Recurrent reciprocal deletions and duplications of 16p13.11: the deletion is a risk factor for MR/MCA while the duplication may be a rare benign variant. *J Med Genet.* (2009) 46:223–32. 10.1136/jmg.2007.055202 18550696 PMC2658752

[6] Heinzen EL Radtke RA Urban TJ Cavalleri GL Depondt C Need AC Rare deletions at 16p13.11 predispose to a diverse spectrum of sporadic epilepsy syndromes. *Am J Hum Genet.* (2010) 86:707–18. 10.1016/j.ajhg.2010.03.018 20398883 PMC2869004

[7] Ingason A Rujescu D Cichon S Sigurdsson E Sigmundsson T Pietiläinen OP Copy number variations of chromosome 16p13.1 region associated with schizophrenia. *Mol Psychiatry.* (2011) 16:17–25. 19786961 10.1038/mp.2009.101 PMC3330746

[8] Ullmann R Turner G Kirchhoff M Chen W Tonge B Rosenberg C Array CGH identifies reciprocal 16p13.1 duplications and deletions that predispose to autism and/or mental retardation. *Hum Mutat.* (2007) 28:674–82. 17480035 10.1002/humu.20546

[9] Tropeano M Ahn JW Dobson RJ Breen G Rucker J Dixit A Male-biased autosomal effect of 16p13.11 copy number variation in neurodevelopmental disorders. *PLoS One.* (2013) 8:e61365. 10.1371/journal.pone.0061365 23637818 PMC3630198

[10] Nagamani SC Erez A Bader P Lalani SR Scott DA Scaglia F Phenotypic manifestations of copy number variation in chromosome 16p13.11. *Eur J Hum Genet.* (2011) 19:280–6. 21150890 10.1038/ejhg.2010.184 PMC3061988

[11] Johnstone M Vasistha NA Barbu MC Dando O Burr K Christopher E Reversal of proliferation deficits caused by chromosome 16p13.11 microduplication through targeting NFkappaB signaling: an integrated study of patient-derived neuronal precursor cells, cerebral organoids and in vivo brain imaging. *Mol Psychiatry.* (2019) 24:294–311. 10.1038/s41380-018-0292-1 30401811 PMC6344377

[12] Takahashi K Tanabe K Ohnuki M Narita M Ichisaka T Tomoda K Induction of pluripotent stem cells from adult human fibroblasts by defined factors. *Cell.* (2007) 131:861–72. 18035408 10.1016/j.cell.2007.11.019

[13] Zhang Y Pak C Han Y Ahlenius H Zhang Z Chanda S Rapid single-step induction of functional neurons from human pluripotent stem cells. *Neuron.* (2013) 78:785–98. 23764284 10.1016/j.neuron.2013.05.029 PMC3751803

[14] Gomes C Smith SC Youssef MN Zheng JJ Hagg T Hetman M RNA polymerase 1-driven transcription as a mediator of BDNF-induced neurite outgrowth. *J Biol Chem.* (2011) 286:4357–63. 10.1074/jbc.M110.170134 21098478 PMC3039349

[15] Sundberg M Tochitsky I Buchholz DE Winden K Kujala V Kapur K Purkinje cells derived from TSC patients display hypoexcitability and synaptic deficits associated with reduced FMRP levels and reversed by rapamycin. *Mol Psychiatry.* (2018) 23:2167–83. 10.1038/s41380-018-0018-4 29449635 PMC6093816

[16] Fujitani M Zhang S Fujiki R Fujihara Y Yamashita T. A chromosome 16p13.11 microduplication causes hyperactivity through dysregulation of miR-484/protocadherin-19 signaling. *Mol Psychiatry.* (2017) 22:364–74. 10.1038/mp.2016.106 27378146 PMC5322274

[17] Martella G Bonsi P Johnson SW Quartarone A. Synaptic plasticity changes: hallmark for neurological and psychiatric disorders. *Neural Plast.* (2018) 2018:9230704. 10.1155/2018/9230704 PMC6218720 30425736

[18] Liu JY Kasperavièiûtë D Martinian L Thom M Sisodiya SM. Neuropathology of 16p13.11 deletion in epilepsy. *PLoS One.* (2012) 7:e34813. 10.1371/journal.pone.0034813 22523559 PMC3327721

[19] de Kovel CG Trucks H Helbig I Mefford HC Baker C Leu C Recurrent microdeletions at 15q11.2 and 16p13.11 predispose to idiopathic generalized epilepsies. *Brain.* (2010) 133(Pt 1):23–32. 19843651 10.1093/brain/awp262 PMC2801323

[20] Mefford HC Muhle H Ostertag P von Spiczak S Buysse K Baker C Genome-wide copy number variation in epilepsy: novel susceptibility loci in idiopathic generalized and focal epilepsies. *PLoS Genet.* (2010) 6:e1000962. 10.1371/journal.pgen.1000962 20502679 PMC2873910

[21] Alkuraya FS Cai X Emery C Mochida GH Al-Dosari MS Felie JM Human mutations in NDE1 cause extreme microcephaly with lissencephaly [corrected]. *Am J Hum Genet.* (2011) 88:536–47. 21529751 10.1016/j.ajhg.2011.04.003 PMC3146728

[22] Bakircioglu M Carvalho OP Khurshid M Cox JJ Tuysuz B Barak T The essential role of centrosomal NDE1 in human cerebral cortex neurogenesis. *Am J Hum Genet.* (2011) 88:523–35. 21529752 10.1016/j.ajhg.2011.03.019 PMC3146716

[23] Tan L Bi B Zhao P Cai X Wan C Shao J Severe congenital microcephaly with 16p13.11 microdeletion combined with NDE1 mutation, a case report and literature review. *BMC Med Genet.* (2017) 18:141. 10.1186/s12881-017-0501-9 29191162 PMC5709987

[24] Paciorkowski AR Keppler-Noreuil K Robinson L Sullivan C Sajan S Christian SL Deletion 16p13.11 uncovers NDE1 mutations on the non-deleted homolog and extends the spectrum of severe microcephaly to include fetal brain disruption. *Am J Med Genet A.* (2013) 161A:1523–30. 10.1002/ajmg.a.35969 23704059 PMC3689850

[25] Houlihan SL Feng Y. The scaffold protein Nde1 safeguards the brain genome during S phase of early neural progenitor differentiation. *Elife.* (2014) 3:e03297. 10.7554/eLife.03297 25245017 PMC4170211

[26] Feng Y Olson EC Stukenberg PT Flanagan LA Kirschner MW Walsh CA. LIS1 regulates CNS lamination by interacting with mNudE, a central component of the centrosome. *Neuron.* (2000) 28:665–79. 10.1016/s0896-6273(00)00145-8 11163258

[27] Feng Y Walsh CA. Mitotic spindle regulation by Nde1 controls cerebral cortical size. *Neuron.* (2004) 44:279–93. 15473967 10.1016/j.neuron.2004.09.023

[28] Pawlisz AS Mutch C Wynshaw-Boris A Chenn A Walsh CA Feng Y. Lis1-Nde1-dependent neuronal fate control determines cerebral cortical size and lamination. *Hum Mol Genet.* (2008) 17:2441–55. 10.1093/hmg/ddn144 18469343 PMC2486443

[29] Lam C Vergnolle MA Thorpe L Woodman PG Allan VJ. Functional interplay between LIS1, NDE1 and NDEL1 in dynein-dependent organelle positioning. *J Cell Sci.* (2010) 123(Pt 2):202–12. 10.1242/jcs.059337 20048338

[30] Sasaki S Shionoya A Ishida M Gambello MJ Yingling J Wynshaw-Boris A LIS1/NUDEL/cytoplasmic dynein heavy chain complex in the developing and adult nervous system. *Neuron.* (2000) 28:681–96. 10.1016/s0896-6273(00)00146-x 11163259

[31] Kalil K Dent EW. Branch management: mechanisms of axon branching in the developing vertebrate CNS. *Nat Rev Neurosci.* (2014) 15:7–18. 10.1038/nrn3650 24356070 PMC4063290

[32] Winden KD Sundberg M Yang C Wafa SMA Dwyer S Chen PF Biallelic mutations in TSC2 lead to abnormalities associated with cortical tubers in human iPSC-derived neurons. *J Neurosci.* (2019) 39:9294–305. 10.1523/JNEUROSCI.0642-19.2019 31591157 PMC6867816

[33] Blattner C Jennebach S Herzog F Mayer A Cheung ACM Witte G Molecular basis of Rrn3-regulated RNA polymerase I initiation and cell growth. *Genes Dev.* (2011) 25:2093–105. 10.1101/gad.17363311 21940764 PMC3197207

[34] Stepanchick A Zhi H Cavanaugh AH Rothblum K Schneider DA Rothblum LI. DNA binding by the ribosomal DNA transcription factor rrn3 is essential for ribosomal DNA transcription. *J Biol Chem.* (2013) 288:9135–44. 23393135 10.1074/jbc.M112.444265 PMC3610986

[35] Mayer C Zhao J Yuan X Grummt I. mTOR-dependent activation of the transcription factor TIF-IA links rRNA synthesis to nutrient availability. *Genes Dev.* (2004) 18:423–34. 10.1101/gad.285504 15004009 PMC359396

[36] Hannan KM Brandenburger Y Jenkins A Sharkey K Cavanaugh A Rothblum L mTOR-dependent regulation of ribosomal gene transcription requires S6K1 and is mediated by phosphorylation of the carboxy-terminal activation domain of the nucleolar transcription factor UBF. *Mol Cell Biol.* (2003) 23:8862–77. 10.1128/MCB.23.23.8862-8877.2003 14612424 PMC262650

[37] Rieker C Engblom D Kreiner G Domanskyi A Schober A Stotz S Nucleolar disruption in dopaminergic neurons leads to oxidative damage and parkinsonism through repression of mammalian target of rapamycin signaling. *J Neurosci.* (2011) 31:453–60. 10.1523/JNEUROSCI.0590-10.2011 21228155 PMC6623444

[38] Kang S Chen X Gong S Yu P Yau S Su Z Characteristic analyses of a neural differentiation model from iPSC-derived neuron according to morphology, physiology, and global gene expression pattern. *Sci Rep.* (2017) 7:12233. 10.1038/s41598-017-12452-x 28947763 PMC5612987

[39] Peek SL Mah KM Weiner JA. Regulation of neural circuit formation by protocadherins. *Cell Mol Life Sci.* (2017) 74:4133–57. 28631008 10.1007/s00018-017-2572-3 PMC5643215

[40] Hill JJ Hashimoto T Lewis DA. Molecular mechanisms contributing to dendritic spine alterations in the prefrontal cortex of subjects with schizophrenia. *Mol Psychiatry.* (2006) 11:557–66. 10.1038/sj.mp.4001792 16402129

[41] Kristiansen LV Beneyto M Haroutunian V Meador-Woodruff JH. Changes in NMDA receptor subunits and interacting PSD proteins in dorsolateral prefrontal and anterior cingulate cortex indicate abnormal regional expression in schizophrenia. *Mol Psychiatry.* (2006) 11:737–47. 10.1038/sj.mp.4001844 16702973

[42] Beneyto M Meador-Woodruff JH. Lamina-specific abnormalities of AMPA receptor trafficking and signaling molecule transcripts in the prefrontal cortex in schizophrenia. *Synapse.* (2006) 60:585–98. 10.1002/syn.20329 16983646

[43] Beneyto M Meador-Woodruff JH. Lamina-specific abnormalities of NMDA receptor-associated postsynaptic protein transcripts in the prefrontal cortex in schizophrenia and bipolar disorder. *Neuropsychopharmacology.* (2008) 33:2175–86. 10.1038/sj.npp.1301604 18033238

[44] Li W Ghose S Gleason K Begovic A Perez J Bartko J Synaptic proteins in the hippocampus indicative of increased neuronal activity in CA3 in schizophrenia. *Am J Psychiatry.* (2015) 172:373–82. 25585032 10.1176/appi.ajp.2014.14010123 PMC4457341

[45] Forsyth JK Lewis DA. Mapping the consequences of impaired synaptic plasticity in schizophrenia through development: an integrative model for diverse clinical features. *Trends Cogn Sci.* (2017) 21:760–78. 10.1016/j.tics.2017.06.006 28754595 PMC5610626

[46] Speese SD Trotta N Rodesch CK Aravamudan B Broadie K. The ubiquitin proteasome system acutely regulates presynaptic protein turnover and synaptic efficacy. *Curr Biol.* (2003) 13:899–910. 10.1016/s0960-9822(03)00338-5 12781128

[47] Bingol B Schuman EM. Activity-dependent dynamics and sequestration of proteasomes in dendritic spines. *Nature.* (2006) 441:1144–8. 10.1038/nature04769 16810255

[48] Hegde AN. The ubiquitin-proteasome pathway and synaptic plasticity. *Learn Mem.* (2010) 17:314–27. 20566674 10.1101/lm.1504010 PMC2904103

[49] Hamilton AM Oh WC Vega-Ramirez H Stein IS Hell JW Patrick GN Activity-dependent growth of new dendritic spines is regulated by the proteasome. *Neuron.* (2012) 74:1023–30. 10.1016/j.neuron.2012.04.031 22726833 PMC3500563

[50] Jarome TJ Helmstetter FJ. The ubiquitin-proteasome system as a critical regulator of synaptic plasticity and long-term memory formation. *Neurobiol Learn Mem.* (2013) 105:107–16. 23623827 10.1016/j.nlm.2013.03.009 PMC3786694

[51] Bousman CA Chana G Glatt SJ Chandler SD Lucero GR Tatro E Preliminary evidence of ubiquitin proteasome system dysregulation in schizophrenia and bipolar disorder: convergent pathway analysis findings from two independent samples. *Am J Med Genet B Neuropsychiatr Genet.* (2010) 153B:494–502. 10.1002/ajmg.b.31006 19582768 PMC4165610

[52] Rubio MD Wood K Haroutunian V Meador-Woodruff JH. Dysfunction of the ubiquitin proteasome and ubiquitin-like systems in schizophrenia. *Neuropsychopharmacology.* (2013) 38:1910–20. 10.1038/npp.2013.84 23571678 PMC3746696

[53] Tasaki T Sriram SM Park KS Kwon YT. The N-end rule pathway. *Annu Rev Biochem.* (2012) 81:261–89. 22524314 10.1146/annurev-biochem-051710-093308 PMC3610525

[54] Nguyen KT Mun SH Lee CS Hwang CS. Control of protein degradation by N-terminal acetylation and the N-end rule pathway. *Exp Mol Med.* (2018) 50:1–8. 10.1038/s12276-018-0097-y PMC6063864 30054456

[55] Burre J. The synaptic function of alpha-synuclein. *J Parkinsons Dis.* (2015) 5:699–713. 26407041 10.3233/JPD-150642 PMC4927875

[56] Kiryk A Sowodniok K Kreiner G Rodriguez-Parkitna J Sönmez A Górkiewicz T Impaired rRNA synthesis triggers homeostatic responses in hippocampal neurons. *Front Cell Neurosci.* (2013) 7:207. 10.3389/fncel.2013.00207 24273493 PMC3823236

[57] Kwon YT Balogh SA Davydov IV Kashina AS Yoon JK Xie Y Altered activity, social behavior, and spatial memory in mice lacking the NTAN1p amidase and the asparagine branch of the N-end rule pathway. *Mol Cell Biol.* (2000) 20:4135–48. 10.1128/MCB.20.11.4135-4148.2000 10805755 PMC85783

[58] Balogh SA Kwon YT Denenberg VH. Varying intertrial interval reveals temporally defined memory deficits and enhancements in NTAN1-deficient mice. *Learn Mem.* (2000) 7:279–86. 10.1101/lm.33500 11040259 PMC311346

[59] Nestler EJ Hyman SE Malenka RC. *Molecular Neuropharmacology : a Foundation for Clinical Neuroscience.* 2nd ed. New York, NY: McGraw-Hill Medical (2009). 498 p.

[60] Nestler EJ Hyman SE. Animal models of neuropsychiatric disorders. *Nat Neurosci.* (2010) 13:1161–9. 20877280 10.1038/nn.2647 PMC3750731

[61] Feldcamp LA Boutros PC Raymond R Fletcher PJ Nobrega JN Wong AHC. Pdxdc1 modulates prepulse inhibition of acoustic startle in the mouse. *Transl Psychiatry.* (2017) 7:e1125. 10.1038/tp.2017.85 28485732 PMC5534953

