# Supplementary material for: 16p13.11 deletion variants associated with neuropsychiatric disorders cause morphological and synaptic changes in induced pluripotent stem cell-derived neurons
Source: Front Psychiatry. 2022 Nov 3;13:924956. doi: 10.3389/fpsyt.2022.924956 (PMC9669751; doi:10.3389/fpsyt.2022.924956)
Supplement: Supplementary file 3 [file Data_Sheet_2.docx]

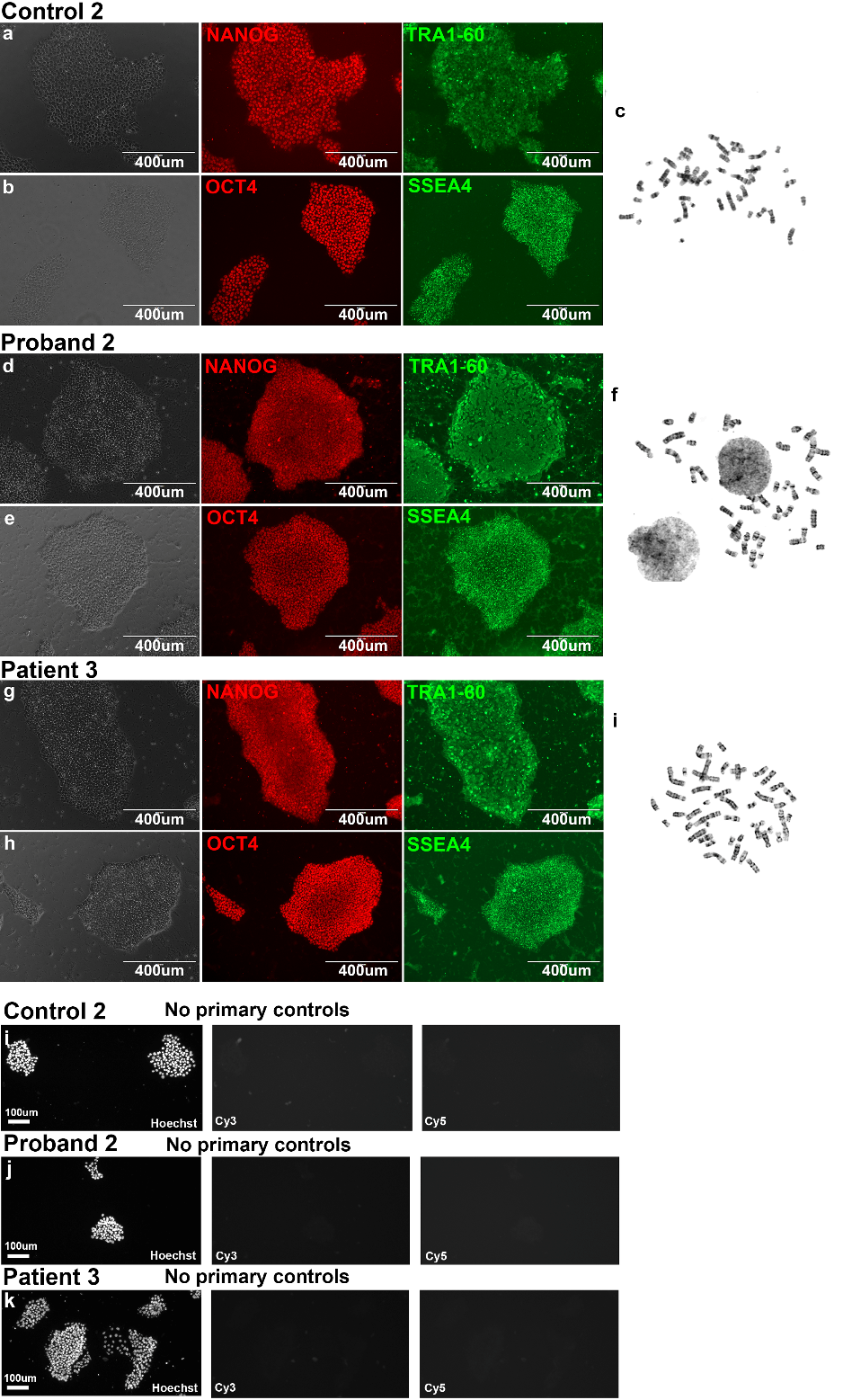


**Supplemental Figure 2. Characterization of iPSCs from family 2**. (**A-B**) Control 2 iPSCs each expressed pluripotency markers, NANOG (**A**, red) and TRA1-60 (**A**, green) as well as OCT4 (**B**, red) and SSEA4 (**B**, green). (**C**) Control 2 iPSCs also had a normal karyotype at the start of the project. (**D-E**) Proband 2 iPSCs each expressed the pluripotency markers NANOG (**D**, red) and TRA1-60 (**D**, green) as well as OCT4 (**E**, red and SSEA4 (**E**, green). (**F**) Proband 2 iPSCs had a normal karyotype at the start of the project. (**G-H**) Patient 3 iPSCs each expressed the pluripotency markers NANOG (**G**, red) and TRA1-60 (**G**, green) as well as OCT4 (**H**, red) and SSEA4 (**H**, green). (**I**) Patient 3 iPSCs had a normal karyotype at the start of the project. Scale bars = 400µm. Pseudo-coloring applied at the time of imaging on the Echo Revolve microscope and not adjusted post-imaging. (**I**-**K**) Control 2 (**I**), Proband 2 (**J**), and Patient 3 (**K**) iPSCs stained with Hoechst and secondary antibodies in Cy3 and Cy5 channels without primary antibodies to show background level from secondary antibodies only. Scale bars for **I - K** = 100µm.
