# Supplementary material for: 16p13.11 deletion variants associated with neuropsychiatric disorders cause morphological and synaptic changes in induced pluripotent stem cell-derived neurons
Source: Front Psychiatry. 2022 Nov 3;13:924956. doi: 10.3389/fpsyt.2022.924956 (PMC9669751; doi:10.3389/fpsyt.2022.924956)
Supplement: Supplementary file 4 [file Data_Sheet_3.docx]

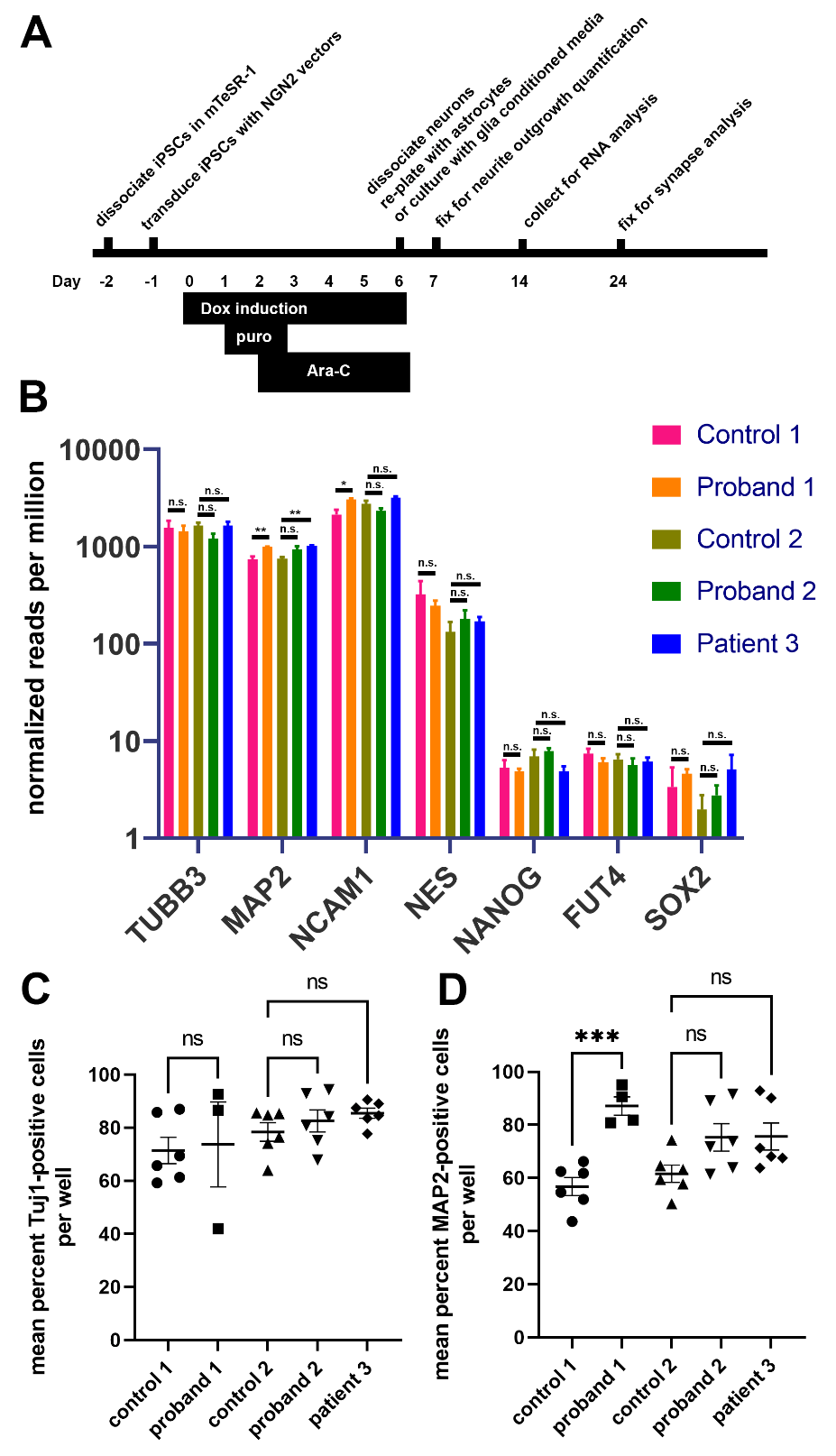


**Supplemental Figure 3. Confirmation of neuronal differentiation via transcriptome and high content imaging data**. (**A**) Schematic of NGN2 differentiation protocol, including timing of phenotypic assays. (**B**) AmpliSeq data from day 14 iPSC-derived neurons revealing increased normalized reads per million (nrpm) expression level data for neuronal genes, *TUBB3*, *MAP2*, and *NCAM1*, low level expression levels of the neural progenitor marker, *NES*, and below confidence level for reliable expression of 10nrpm of pluripotency markers, *NANOG*, *FUT4*, *SOX2*. Data are shown as ±SEM, n=3 differentiations, n.s.= not significant, *p<0.05, **p<0.01. Note: Scale on y-axis is log10. (**C**-**D**) Quantification of high content imaging from day 7 iPSC-derived neurons revealing mean percent Tuj1-positive (**C**) and MAP2-positive (**D**) cells per well. Data are shown as ±SEM, each data point represents a mean well value averaged from 9 wells per field across 3 differentiations, ns= not significant, ***p<0.005.
