# Supplementary material for: 16p13.11 deletion variants associated with neuropsychiatric disorders cause morphological and synaptic changes in induced pluripotent stem cell-derived neurons
Source: Front Psychiatry. 2022 Nov 3;13:924956. doi: 10.3389/fpsyt.2022.924956 (PMC9669751; doi:10.3389/fpsyt.2022.924956)
Supplement: Supplementary file 5 [file Data_Sheet_4.docx]

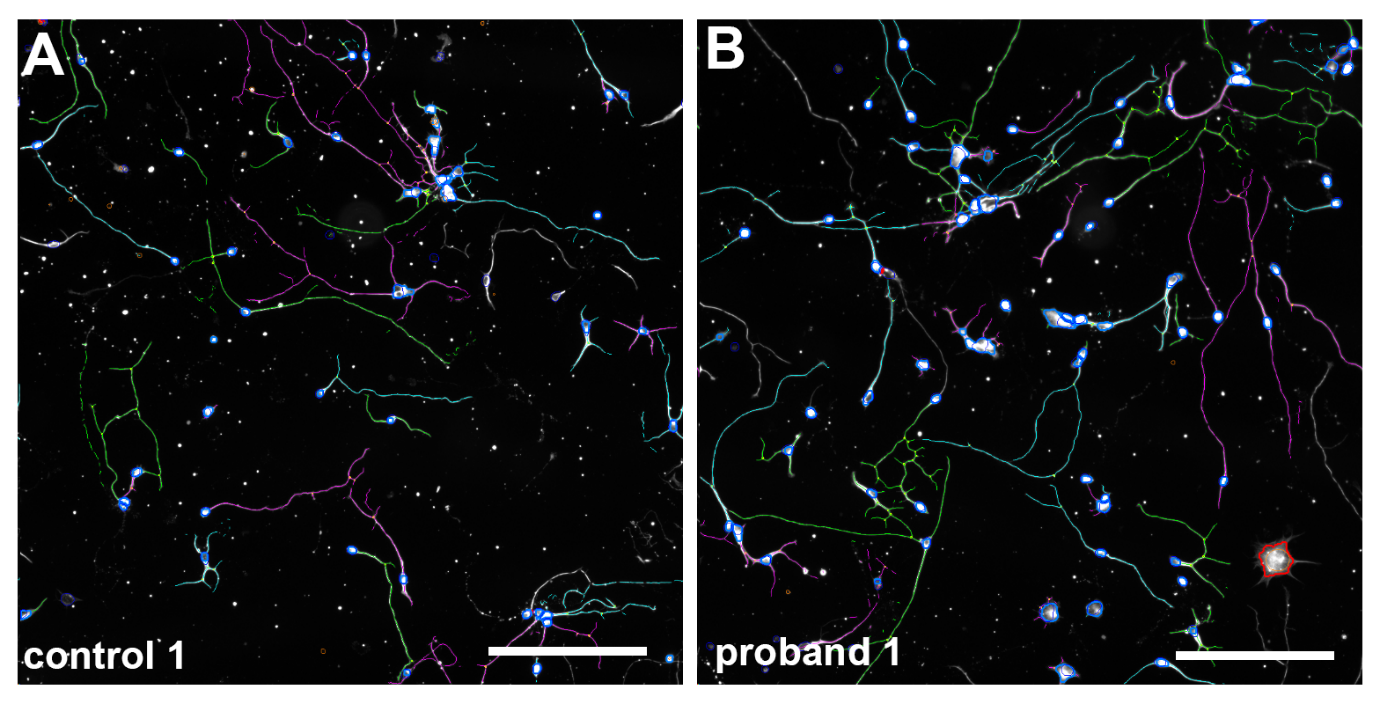


**Supplemental Figure 4. Neurite mask algorithm example.** Representative images from control 1 (**A**) and proband 1 (**B**) neurons showing the neurite quantification algorithm that was used to select neuronal cell bodies (blue) and neurites (pink and green), while not quantifying non-neuronal cells (red). Neurites are traced with different colors to identify individual cells. Scale bars = 300µm.
