# Supplementary material for: 16p13.11 deletion variants associated with neuropsychiatric disorders cause morphological and synaptic changes in induced pluripotent stem cell-derived neurons
Source: Front Psychiatry. 2022 Nov 3;13:924956. doi: 10.3389/fpsyt.2022.924956 (PMC9669751; doi:10.3389/fpsyt.2022.924956)
Supplement: Supplementary file 6 [file Data_Sheet_5.docx]

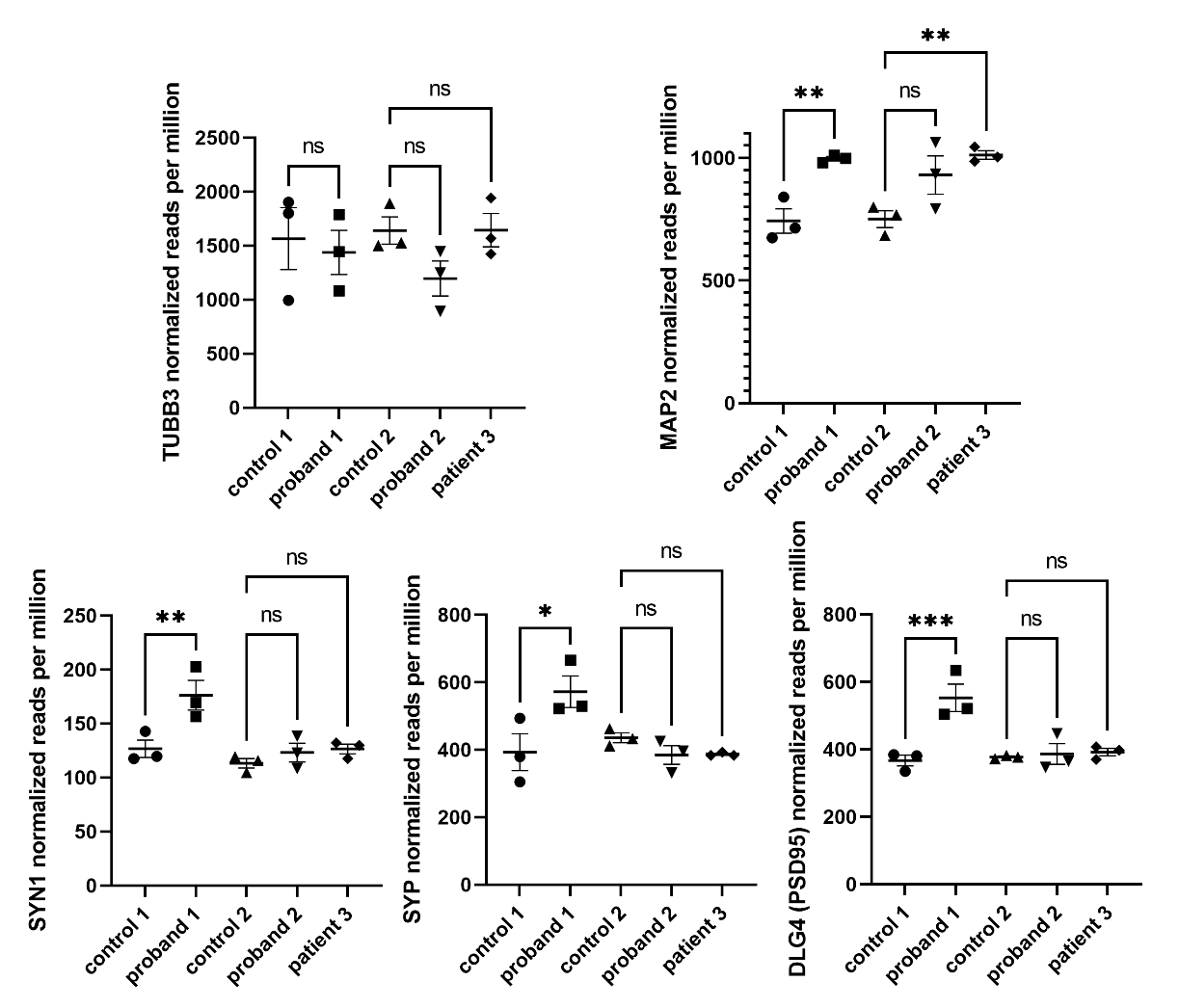


**Supplemental Figure 5.** Quantification of synaptic genes via transcriptomic analysis. AmpliSeq data from day 14 iPSC-derived neurons revealing normalized reads per million values for *SYN1*, *SYP*, and *DLG4*, the markers that are also used for synaptic staining. Data are shown as ±SEM, n=3 differentiations, ns= not significant, *p<0.05, **p<0.01, ***p<0.005.
