# Supplementary material for: 16p13.11 deletion variants associated with neuropsychiatric disorders cause morphological and synaptic changes in induced pluripotent stem cell-derived neurons
Source: Front Psychiatry. 2022 Nov 3;13:924956. doi: 10.3389/fpsyt.2022.924956 (PMC9669751; doi:10.3389/fpsyt.2022.924956)
Supplement: Supplementary file 7 [file Data_Sheet_6.docx]

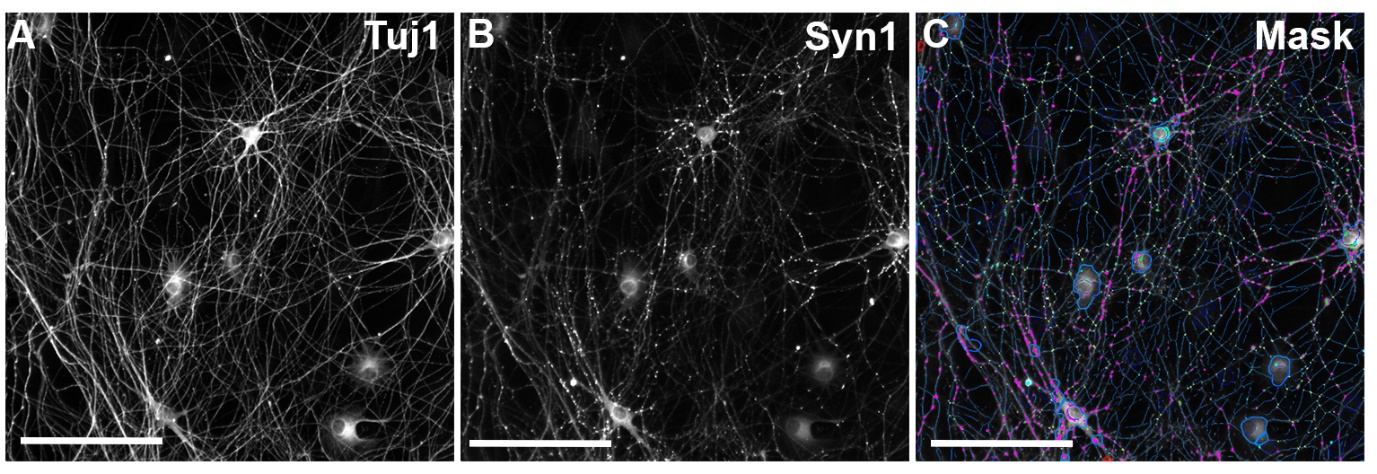


**Supplemental Figure 6. Synapse mask algorithm example.** Representative image of control 1 iPSC-derived neurons stained with Tuj1 (**A**), Synapsin1 (Syn1, **B**), and Mask (**C**) showing the synapse quantification algorithm with cell bodies (blue circles), neurites (blue lines), and synapses (pink dots) identified. Scale bars = 150µm.
