# Supplementary material for: 16p13.11 deletion variants associated with neuropsychiatric disorders cause morphological and synaptic changes in induced pluripotent stem cell-derived neurons
Source: Front Psychiatry. 2022 Nov 3;13:924956. doi: 10.3389/fpsyt.2022.924956 (PMC9669751; doi:10.3389/fpsyt.2022.924956)
Supplement: Supplementary file 8 [file Data_Sheet_7.docx]

**
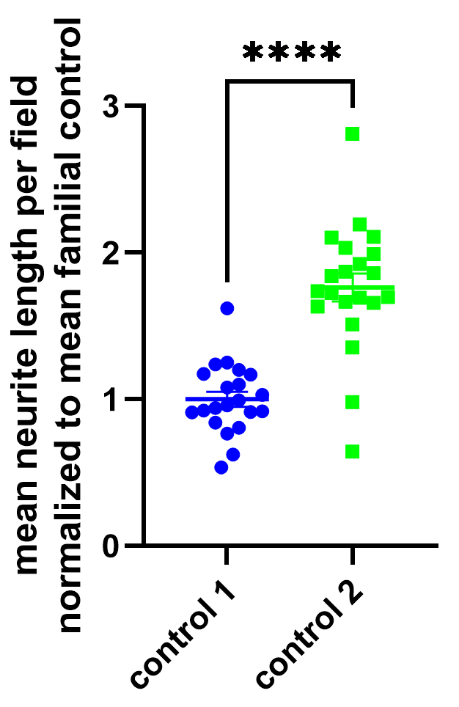
**

**Supplemental Figure 7. Comparison of neurite outgrowth in control 1 and control 2.** Quantification of neurite outgrowth in cultures used to quantify synapse number reveal a significant decrease in mean neurite outgrowth per field in control 1 compared to control 2. Data are shown as ±SEM, each data point represents a mean well value averaged from 9 fields per well across 3 differentiations, **** p<0.0001.
