# Supplementary material for: 16p13.11 deletion variants associated with neuropsychiatric disorders cause morphological and synaptic changes in induced pluripotent stem cell-derived neurons
Source: Front Psychiatry. 2022 Nov 3;13:924956. doi: 10.3389/fpsyt.2022.924956 (PMC9669751; doi:10.3389/fpsyt.2022.924956)
Supplement: Supplementary file 9 [file Data_Sheet_8.docx]

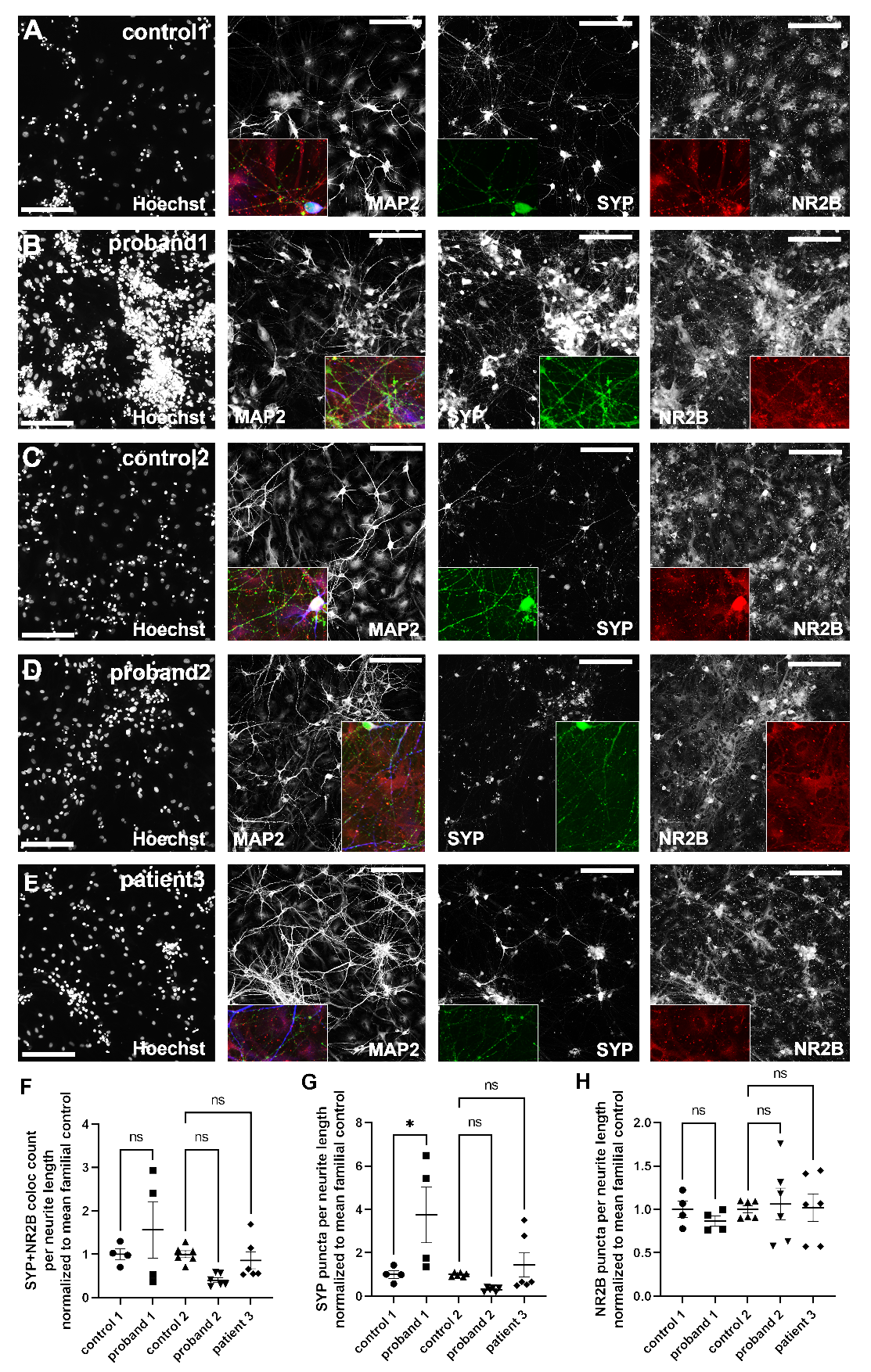


**Supplemental Figure 8**. No change in glutamatergic synapse count in 16p13.11 deletion neurons. Representative images of control 1 (**A**), proband 1 (**B**), control 2 (**C**), proband 2 (**D**), and patient 3 (**E**) day 24 NGN2 neurons co-cultured with astrocytes and immunostained for MAP2, Synaptophysin (SYP), and NR2B-glutamate receptors. Images were captured at 20X. (**F**) Quantification of average SYP+NR2B colocalized puncta per neurite length. (**G**) Quantification of SYP puncta per neurite length. (**H**) Quantification of NR2B puncta per neurite length. Scale bars = 150µm. (**F-H**) data are shown as ±SEM, each data point represents a mean well value averaged from 9 fields per well across 3 differentiations, ns=not significant, *p<0.05. Pseudo-coloring was applied to allow for merged images.
